# DIAMONDS—a diabetes self-management intervention for people with severe mental illness: protocol for an individually randomised controlled multicentre trial

**DOI:** 10.1136/bmjopen-2024-090295

**Published:** 2025-03-27

**Authors:** Grace Catherine O’Carroll, Jennifer V E Brown, Claire Carswell, Charlie Peck, Gregor Russell, R A Ajjan, Jan Rasmus Boehnke, Peter A Coventry, Michelle Hadjiconstantinou, Catherine Hewitt, Richard Ian Gregory Holt, Vicki Johnson, Ian Kellar, Jinshuo Li, Laura Mandefield, David Osborn, Steve Parrott, Lucy Sheehan, David Shiers, Judith Watson, Najma Siddiqi, DIAMONDS Research Team, Rowena Jacobs

**Affiliations:** 1 Department of Health Sciences, University of York, York, UK; 2 Bradford District Care NHS Foundation Trust, Saltaire, Bradford, UK; 3 Leeds Institute for Cardiovascular and Metabolic Medicine, University of Leeds, Leeds, UK; 4 School of Health Sciences, University of Dundee, Dundee, UK; 5 Diabetes Research Centre, University of Leicester, Leicester, UK; 6 NIHR Biomedical Research Centre, University of Leicester, Leicester, UK; 7 Human Development and Health Academic Unit, University of Southampton, Southampton, UK; 8 Southampton National Institute for Health Research Biomedical Research Centre, University Hospital Southampton NHS Foundation Trust, Southampton, UK; 9 Leicester Diabetes Centre, University Hospitals of Leicester NHS Trust, Leicester, UK; 10 School of Psychology, The University of Sheffield, Sheffield, UK; 11 Division of Psychiatry, University College London, London, UK; 12 Psychosis Research Unit, Greater Manchester Mental Health NHS Foundation Trust, Manchester, UK; 13 School of Medicine, Keele University, Newcastle-under-Lyme, UK; 14 Hull York Medical School, University of York, York, UK

**Keywords:** Self-Management, Diabetes Mellitus, Type 2, MENTAL HEALTH

## Abstract

**Introduction:**

Type 2 diabetes mellitus (T2DM) is two to three times more common in people with severe mental illness (SMI) than in the general population. Supporting self-management in diabetes is fundamental to improving clinical outcomes. The DIAMONDS trial aims to evaluate the clinical and cost effectiveness of a novel, codesigned, supported diabetes self-management programme for people with T2DM and SMI.

**Methods and analysis:**

This multicentre, two-armed, parallel, individually randomised controlled trial will be conducted in National Health Service mental health trusts across England. We will recruit 380 participants (≥18 years old) with a diagnosis of SMI (schizophrenia, bipolar disorder, schizoaffective disorder, psychosis and severe depression) and T2DM. Eligible and consenting participants will be randomised to the DIAMONDS intervention or treatment as usual. The intervention group will receive one-to-one sessions with a trained DIAMONDS Coach for six months. These sessions will focus on goal setting, action planning and diabetes self-management education, supported by a paper-based workbook and an optional digital application. Individuals allocated to the control group will continue to receive usual care and may be offered National Institute for Health and Care Excellence-recommended generic diabetes self-management education programmes in line with usual practice. The primary outcome is the difference in glycated haemoglobin (HbA1c) between both groups at 12 months postrandomisation. The secondary outcomes include measures of physical and mental health, diabetes complications and physical activity. Economic and process evaluations will also be performed. Outcomes will be collected at baseline and at six and 12 month post-randomisation.

**Ethics and dissemination:**

This study received ethics approval by the West of Scotland Research Ethics Committee 3 (22/WS/0117). Findings will be published in peer-reviewed, academic and professional journals. We will also be producing plain language summaries, infographics and audio summaries on the website, as well as attending conferences and dissemination events. A summary of the results will be distributed to all participants and other relevant stakeholders, and we will use social media channels, websites and knowledge exchange events to communicate our findings beyond academic audiences.

**Trial registration number:**

ISRCTN22275538.

**STRENGTHS AND LIMITATIONS OF THIS STUDY**

- The DIAMONDS randomised controlled trial (RCT) follows on from a feasibility study (DIAMONDS Feasibility Study, ISRCTN15328700), which confirmed the acceptability and feasibility of the intervention and allowed us to make important changes to some study processes before commencing with this full-scale RCT.
- The trial incorporates Urdu language materials to improve inclusivity.
- The trial includes a mixed-methods process evaluation and economic evaluation.
- The primary outcome (glycated haemoglobin: HbA1c) is measured from blood samples analysed at a central masked laboratory and is therefore unlikely to be affected by participants not being masked to their group allocation.
- Participants will know whether they receive the intervention or not, which could impact their responses for patient-reported outcomes or involvement in the trial.

## Introduction

 People with severe mental illness (SMI; ie, long-term mental illnesses such as schizophrenia, schizoaffective disorder, bipolar disorder and severe depression)^1^ experience higher rates of physical illness than the general population. Their life expectancy is 15–20 years shorter^2^,^5^ mainly due to comorbid physical illnesses.^6^,^8^ Accessing clinically and cost-effective healthcare for individuals with a combination of mental and physical illness is recognised as challenging. The symptoms and the pharmacological treatments of SMI and physical illness can negatively interact, leading to higher illness and treatment burden compared with the general population.^9^ The resulting health inequalities are especially apparent when SMI is comorbid with diabetes. Type 2 diabetes mellitus (T2DM) is two to three times more common in people with SMI than in the general population^5 10^ and is associated with poorer outcomes than those seen in individuals with diabetes alone.^6^,^8^

Supporting self-management in diabetes, in common with other long-term conditions (LTCs), is fundamental to improving clinical outcomes,^11^,^13^ as most diabetes care falls to self-management.^14^ Self-management refers to the skills, practices and behaviours that a person engages in to protect and promote their health. Diabetes self-management activities include improving diet; physical activity; smoking cessation; monitoring blood glucose levels; preventing complications and treatment adherence.^15 16^ ‘Self-management education’ is key to supporting self-management.^13 17 18^ In England, diabetes self-management education and support programmes are recommended for recently diagnosed persons and their family members or supporters.^13^ Such programmes typically include educational and behavioural elements to increase knowledge, skills and capacity for self-management.^19 20^ Self-management education programmes for the general population with diabetes have been found to be clinically and cost-effective.^1419 21^,^24^

For people with SMI and diabetes, self-management support is rarely offered (although reliable data on this are difficult to obtain).^25^ Moreover, the effectiveness of diabetes self-management programmes for this population is largely unknown as research typically excludes them.^26^,^28^ SMI is characterised by disturbances of thought, perception, affect and motivation,^29 30^ which influence self-efficacy, literacy, lifestyle, behaviour and family life.^31^,^34^ Diabetes self-management programmes designed for the general population do not address these important barriers,^35^,^38^ and programmes specifically for people with SMI do not currently exist. The STEPWISE trial^39^ tested a group structured lifestyle education programme to support weight reduction in people with schizophrenia. While the intervention was neither clinically nor cost-effective, the STEPWISE trial aimed to overcome the unacceptable health inequalities among people with SMI and highlighted the challenges of improving physical health in people with schizophrenia.^12^

## Aim and objectives

The DIAMONDS randomised controlled trial (RCT) aims to investigate the clinical and cost-effectiveness of a self-management intervention for people with SMI and T2DM compared with usual care. We will conduct an economic evaluation to assess the cost-effectiveness of the DIAMONDS intervention and a process evaluation that will address questions about whether the intervention was delivered as intended and how outcomes were determined. An intervention fidelity assessment will also be undertaken. This paper describes the trial protocol.

### Primary outcome

The primary outcome is the adjusted difference in glycated haemoglobin (HbA1c) between the groups at 12 months postrandomisation. To avoid the inadvertent introduction of differences in measurements of HbA1c through the use of several local laboratories, one central United Kingdom Accreditation Service registered laboratory will be used for all blood sample analyses. Blood samples will be sent to the laboratory from the participating sites. The laboratory will return test results (recorded as mmol/mol and %) to the study team at the University of York (UoY).

### Secondary outcomes

Secondary outcomes were selected to allow for a broad clinical and psychosocial profile as well as to cover domains of the core outcome set for trials evaluating such interventions in this population.

Outcomes include measures of physical health (total cholesterol, haemoglobin, body mass index, waist circumference, blood pressure, smoking status and urinary albumin to creatinine ratio), physical activity (recorded with an accelerometer and participant self-report), mental health, diabetes measures, quality of life, health resource use and mechanisms of action (MoA). Full details of the outcome measures can be found in online supplemental appendix 1.

## Methods and analysis

This protocol is reported in line with the SPIRIT (Standard Protocol Items: Recommendations for Interventional Trials) checklist.^40^ See online supplemental appendix 2 for a copy of the completed checklist.

### Trial design

The DIAMONDS trial is a multicentre, two-armed, parallel, individual RCT with embedded process and economic evaluations. The trial includes a 12 month internal pilot phase to assess recruitment assumptions and optimise trial processes (full details of the pilot phase can be found in online supplemental appendix 3). Participants will be followed up for one year with outcome assessments conducted at six and 12 months post-randomisation. The overall study is planned to start in September 2022 and finish in September 2025. Recruitment is planned to start in December 2022 and finish in September 2024.

### Setting and recruitment

The study setting will include National Health Service (NHS) mental health trusts, general practices acting as Participant Identification Centres (PICs) and third sector organisations providing support to individuals with SMI and/or diabetes across England. Participants from previous studies who have provided consent will also be contacted, and individuals will be able to self-refer into the trial. An up-to-date list of recruiting sites is available on the DIAMONDS website (https://www.diamondscollaboration.org.uk/).

Participants will be recruited using methods successfully deployed in the DIAMONDS feasibility study,^41^ using a staged consent procedure. All participant-facing documents were produced in collaboration with DIAMONDS Voice (https://www.diamondscollaboration.org.uk/diamonds-voice), the service user and carer group that has been an integral part of the DIAMONDS programme for several years. Participants will be recruited from these sources and settings:

#### NHS mental health trusts

Authorised research and development (R&D) staff at participating secondary care sites will run searches in databases and screen community mental health team (CMHT) caseloads for potentially eligible patients using the eligibility criteria outlined below. There may also be direct referrals from consultants/CMHTs or through discharge meetings conducted with inpatient wards.

Potential participants will receive a study information pack containing an invitation letter and a short patient information sheet (PIS) and will have the chance to discuss any questions they have with the research team (in person or over the phone). Following this, they will receive the full PIS. These potential participants will be contacted a few days later to arrange a face-to-face meeting with the research team, where there will be a further opportunity to ask questions relating to the study. If the individual wants to take part in the trial, they will be asked to give written informed consent (see online supplemental appendix 4 for a copy of the consent form).

#### GP database screening

General practices will be asked to consult their SMI and LTC quality and outcomes framework registers to screen for potentially eligible patients using the inclusion criteria outlined below. General practitioners (GPs) at participating practices will check the lists produced by the database search to confirm eligibility. They will also approach potential participants at their annual health checks. Eligible patients will initially receive study information documents from their practice, usually via mailout to their home address. Where staff capacity allows, PIC sites will follow this up with a phone call. Consent-to-contact (CTC) will be obtained from interested patients, either via return of a CTC form or verbally during the follow-up phone call and passed on to appropriate research teams at mental health trusts who will then follow the same recruitment process as described above.

#### Identification of potential participants from existing research cohorts

Individuals who have previously taken part in related research projects conducted within our research group at UoY and who have given permission to be approached about future opportunities to participate in research will be contacted. Individuals identified via this route will receive the study information documents and will return a CTC form if interested. This CTC form will be passed on to the research teams at the mental health trusts and follow the process described above.

#### Recruitment from third sector and service user groups

We will work with relevant local third sector organisations and service user groups. Individuals who are interested in taking part in the trial will be directed to the person in the organisation/service supporting the trial, or the DIAMONDS study team. They will be provided with a short PIS and asked to complete and return a CTC form. The recruitment process will continue as previously described.

### Eligibility criteria

Participants must (1) be aged 18 years or older and living in the community; (2) have any of the following SMI diagnoses: schizophrenia, bipolar disorder, schizoaffective disorder, psychosis, severe depression and (3) have T2DM. The diagnosis of SMI and T2DM must be confirmed by a clinician or be stated in the patient medical records.

People will be excluded if they: (1) have cognitive impairments that would preclude the individual from participation in the trial and engagement with the intervention; (2) have gestational diabetes; (3) have type 1 diabetes; (4) have other types of secondary diabetes; (5) lack capacity to consent to participate in the trial as defined by the 2005 Mental Health Capacity Act or (6) are currently in an inpatient stay in an acute or mental health hospital.

### Patient pathway

**Figure 1:**
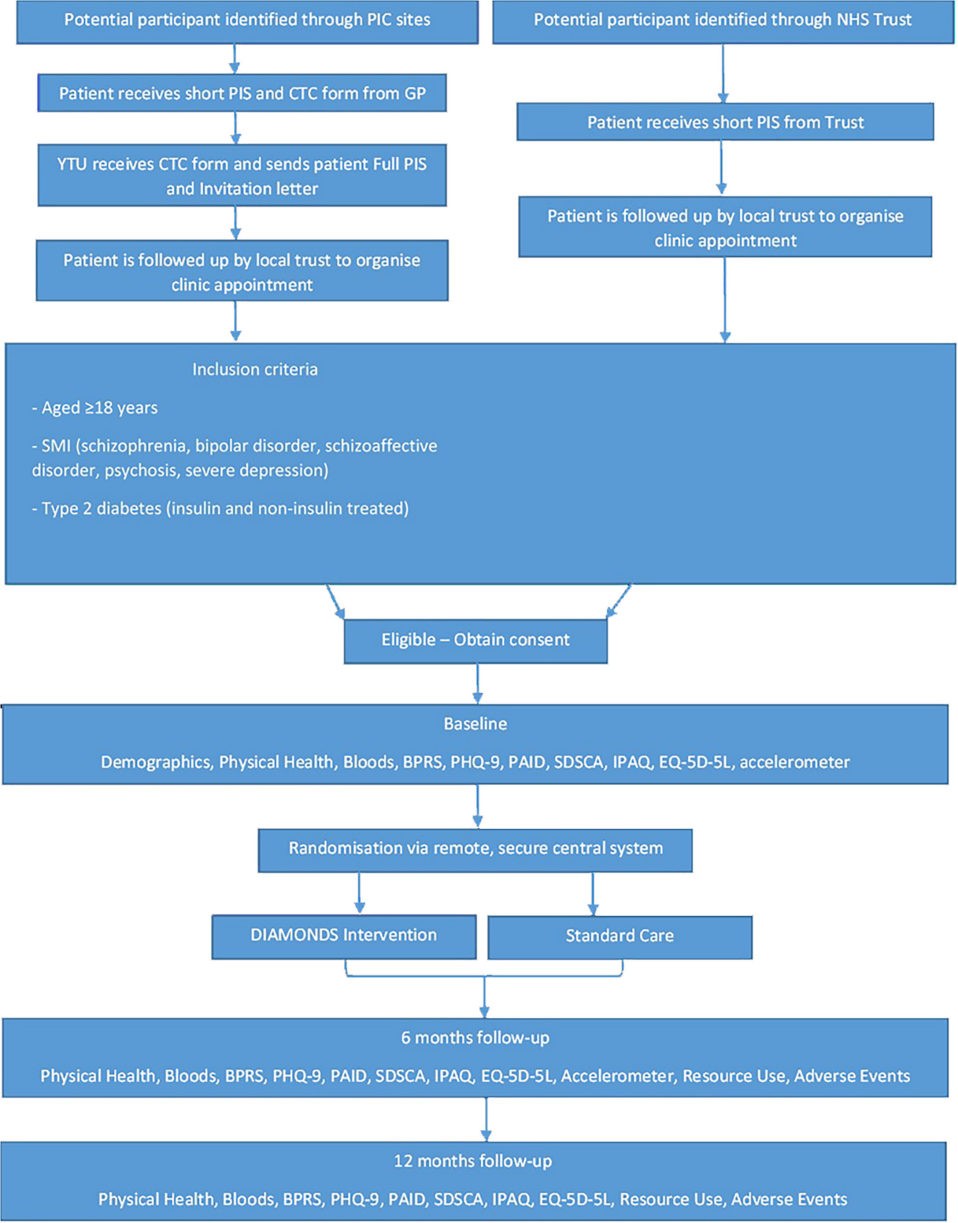
Participant pathway. BPRS, Brief Psychiatric Rating Scale^60^; CTC, consent-to-contact; GP, general practitioners; EQ-5D-5L, EuroQol 5 Dimension 5 Level^61^; IPAQ, International Physical Activity Questionnaire; NHS, National Health Service; PAID, Problem Areas in Diabetes Scale^62^; PHQ-9, Patient Health Questionnaire-9^63^; PIC, Participant Identification Centres; SDSCA, Summary of Diabetes Self-Care Activities Scale^64^; YTU, York Trials Unit.

Figure 1 illustrates the participant pathway through the trial.

### Assignment of groups

Eligible and consenting participants will be randomised on a 1:1 basis to the DIAMONDS intervention or usual care using computer-generated permuted blocks of randomly varying size. York Trials Unit (YTU) will provide a central web-based randomisation service for R&D teams at sites to use when assigning participant allocations. Participants will then be informed of their allocation during their baseline visit or shortly following.

### Blinding

Efforts will be made to ensure R&D staff responsible for data collection remain blinded to treatment allocation. Should a participant inadvertently reveal their allocation to an outcome assessor, or the assessor becomes unblinded for any reason, this will be recorded in the outcome assessment case report form (CRF) at the relevant time.

Designated R&D staff will be tasked with randomising participants and coordinating the handover of participants in the intervention group to a DIAMONDS Coach. Due to the nature of the comparison between the DIAMONDS intervention and treatment as usual, neither participants themselves nor the intervention facilitators (DIAMONDS Coaches) will be blinded.

The trial statisticians and health economists will not be blinded.^42^ The DIAMONDS Programme Manager and Trial coordinators will remain unblinded and will not be involved in the analysis of data.

### The DIAMONDS intervention

The DIAMONDS intervention was co-designed with service users, carers, members of the service user and carer group DIAMONDS Voice and healthcare professionals^43^ and is a tailored self-management support intervention to help people with T2DM and SMI self-manage diabetes through:

- Increasing knowledge and skills for diabetes self-management.
- Providing support to increase their physical activity levels and make healthier food choices.
- Identifying and addressing sleep difficulties, barriers to taking medications and other key problem areas as identified by the participant with support from their Coach, a healthcare professional who has been trained in the delivery of the DIAMONDS intervention.
- Supporting participants to manage their diabetes within the context of fluctuating and low mood.

The acceptability of the intervention to participants and DIAMONDS Coaches was confirmed in the DIAMONDS feasibility study. Prior to the start of the RCT, we refined the intervention in line with findings from the feasibility study, which will be reported elsewhere (DIAMONDS Feasibility Study, ISRCTN15328700). A brief summary of the findings can be found (https://www.hra.nhs.uk/planning-and-improving-research/application-summaries/research-summaries/diamonds-feasibility-study-v10/).

**Figure 2:**
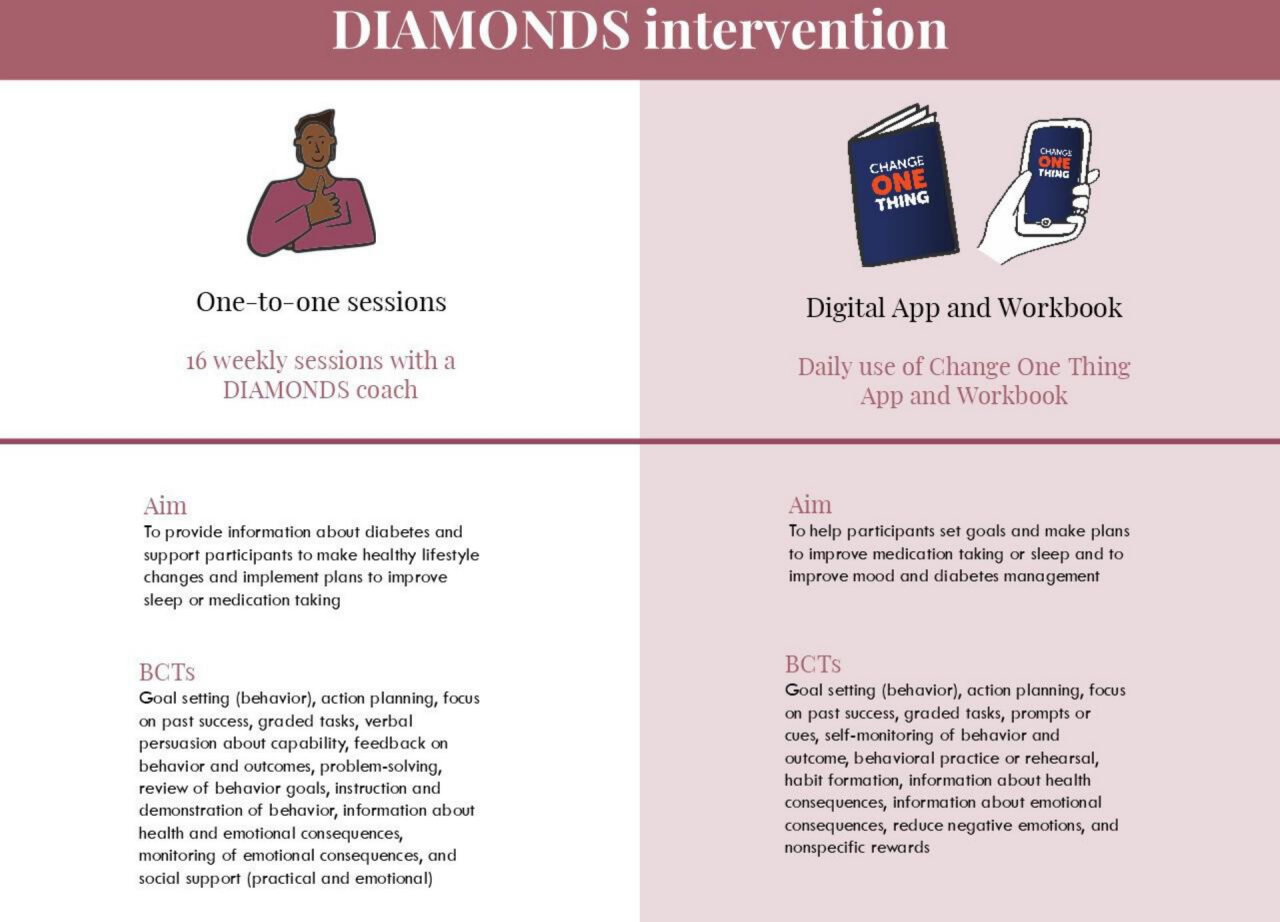
DIAMONDS intervention. BCTs, Behaviour Change Techniques.

The intervention will be delivered by a DIAMONDS Coach over a period of 6 months, using a combination of individual sessions and daily use of a paper-based workbook (the ‘DIAMONDS Workbook’) which can be supported by daily use of a digital app (‘Change One Thing’; optional) (see figure 2 and details below).

If the participant wishes to stop receiving the intervention before the end of the six months, the Coach will still support participants to set longer term goals and action plans for self-management and help them to access appropriate support to implement these, as is done for participants who complete the six month intervention period. Participants will be able to continue engaging with intervention content after follow-up data are collected through continued use of the app and/or workbook. Other reasons for the discontinuation of the intervention would be the death of the participant or if they remain an inpatient that takes them beyond the six month mark.

Participants in the intervention will be permitted to continue with current care alongside the intervention.

### Control group

Participants in the control group will access usual care for people with SMI and diabetes. This will include primary care health checks for SMI and diabetes along with community-based mental healthcare through CMHTs. Participants in the control group will be eligible to self-enrol in existing programmes. Participants randomised to the control group will be signposted to these services immediately following randomisation.

### Data collection and management

Data will be collected at baseline, six and 12 months post-randomisation during appointments with the R&D teams at participating sites taking place either on Trust premises or at the participant’s home. The blood samples for the primary (HbA1c) and secondary (haemoglobin and cholesterol) outcomes will be collected by an appropriately trained member of staff and will be sent to a central laboratory for analysis. The other secondary outcomes will be collected through paper-based CRFs which will be returned to YTU and then scanned using specialist software. The data will be checked against the hard copy of the CRF, error checked and validation checks run against the database. Queries will be raised with the site if discrepancies are identified during validation or on receipt. All training for completing CRFs will be conducted during site set-up and will be recorded on a delegation and training log. There will be a range of centralised monitoring activities (eg, eligibility, consent and safety checks) undertaken as well as being in regular contact with sites to discuss any issues encountered. The full data collection timetable is outlined in online supplemental appendix 5.

To gain objective measures of physical activity in addition to self-report questionnaire data, participants will be asked to wear a wrist-worn accelerometer (GENEActiv, Activinsights, Kimbolton, UK) for seven days at baseline and six months follow-up. Accelerometer data will not be collected during the 12 month follow-up due to previously reported decreased adherence levels following six months.^39^ The devices are blinded, that is, participants will not be able to see or interact with their data during the wear period.

Each participant will be offered a £10 high street gift voucher at their baseline, six and 12 months appointments.

#### Confidentiality and data protection

Each participant will be allocated a unique trial identification number. This number will be used to identify participants throughout the study. Data will be held according to the General Data Protection Regulations and the UK Policy Framework for Health and Social Care.^44^ Anonymised trial data will be securely archived by the UoY for a minimum of 10 years. Personal data of participants will be stored for up to three years after the study has ended for the purpose of disseminating study findings. Full details of the data protection regulations are outlined in online supplemental appendix 6.

### Patient and public involvement (PPI)

During development and throughout the trial, we have been collaborating with DIAMONDS Voice, a service-user and carer group dedicated to supporting this work. The group consists of adults with SMI as well as family carers. DIAMONDS Voice members have contributed critically to the intervention content as well as the development of the intervention materials (app and workbook). For this RCT, they reviewed all participant-facing documentation, including consent forms, invitation letters and questionnaires, and were consulted about the acceptability of taking blood and undertaking measurements of their physical health. They continue to advise on recruitment strategies and will support recruitment within their own networks as appropriate and feasible. Members of DIAMONDS Voice will also be involved in the dissemination of trial findings and wider knowledge exchange activities.

### Sample size and statistical analysis

#### Sample size

The sample size calculation was based on detecting a clinically meaningful difference of 5.5 mmol/mol (0.5%) in HbA1c at 12 months. This difference was selected based on data from trials of diabetes self-management in the general diabetes population^45 46^ and National Institute for Health and Care Excellence (NICE) guideline on T2DM management.^13^ For approximately 90% power, at the 5% significance level, assuming an average cluster size of 10–12 participants per DIAMONDS Coach with an intraclass correlation of 0.02 in the intervention group and adjusting for 20% attrition, it was estimated that 450 participants need to be randomised, with 225 per group.

Owing to slower than anticipated recruitment, we discussed options to revise the target sample size with the Programme Steering Committee (PSC) in February 2024. With the approval of the PSC, we amended the sample size by including an adjustment for the correlation between baseline and 12 months HbA1c (0.3) to reflect the repeated measures analysis model planned for the primary analysis. This led to a reduction in target sample size to 380 participants; statistical power at 88% is retained and all other assumptions remain the same.

#### Statistical analysis

Full analyses will be detailed in a statistical analysis plan (SAP), which will be finalised and made available before the end of data collection. Statistical analyses will be on an intention to treat basis and statistical significance will be at the 5% level (unless otherwise stated in the SAP). Analyses will be conducted in the latest available version of Stata or similar statistical software. Baseline characteristics will be reported descriptively by treatment group. Continuous data will be summarised as means, SD, medians and ranges and categorical data will be summarised as frequencies and percentages. Data will be visually inspected and any imbalance reported. No interim analyses will be conducted.

##### Primary outcome

HbA1c at 12 months post-randomisation will be analysed using a mixed-effects regression analysis, with HbA1c values at six and 12 months follow-up as the dependent variables. Baseline HbA1c values, randomised treatment group, time, and a treatment group-by-time interaction, as well as other important baseline covariates will be included as fixed effects, and the DIAMONDS Coach who delivered the intervention will be included as a random effect, nested within treatment group.

##### Sensitivity analyses

The amount of missing data will be reported for each randomised group, and we will also compare the baseline characteristics of participants who are included in the primary analysis to ensure that any missing data have not produced any imbalance in the groups in important covariates. The amount of missing data will be mitigated by including all data in the primary analysis model, which allows the inclusion of any patient with complete baseline data and valid outcome data at one or more follow-up points. Complier Average Causal Effect (CACE)^47^ analyses will be performed for the primary outcome to assess the impact of compliance with the intervention on treatment estimates.

##### Subgroup analyses

A subgroup analysis will be performed to explore any differential treatment effects for different levels of HbA1c at baseline.^48^ We will also conduct exploratory subgroup analysis by ethnicity and by insulin use status. The results of any subgroup analysis will be treated cautiously, detailed in advance in the SAP and include hypothesised direction of effect, in line with best practice.^49^

##### Secondary outcomes

Secondary outcomes relating to participants’ physical health, mental health and diabetes measures will be analysed using mixed-effects regression analysis for continuous outcomes and logistic mixed models for categorical outcomes. Models will include assessments at all available time-points and will provide an overall treatment effect over 12 months, as well as estimates at individual time-points (six and 12 months), reported as estimates and 95% CIs. Accelerometer data will be collected at baseline and six months post-randomisation. Data will be analysed using the R-package GGIR,^50^ which performs signal processing of the raw data, including auto-calibration, detection of abnormal values, detection of non-wear and calculation of the average magnitude of dynamic acceleration (Euclidean norm minus one g (ENMO)). Descriptive statistics for accelerometer data will be reported for each treatment group at each time point (baseline and six months) and differences between treatment groups will be reported, adjusted for baseline.

### Process evaluation

The process evaluation will draw on a mixed-methods approach, harnessing data from both qualitative and quantitative sources to address questions about whether the intervention was delivered as intended (ie, fidelity) and how outcomes were produced (ie, MoAs). Additionally, the process evaluation will aim to identify contextual and service-level barriers and enablers to post-trial implementation and scale-up, including whether the intervention can support self-management of other LTCs in people with SMI. Drawing on best practice methodology for process evaluations,^51^ we will identify and assess key dimensions related to what intervention activity and content was delivered and how.

#### Intervention fidelity observations

In accordance with the guidance set out by Bellg (2004),^52^ the Intervention Fidelity (IF) framework for the DIAMONDS RCT will measure: (1) adherence (whether the content of the intervention sessions was delivered as it was designed); (2) quality of delivery of intervention sessions (use of Behaviour Change Techniques and the manner/behaviour in which the Coach delivers the programme); (3) duration (mean, SD and range) of intervention sessions and (4) dose (number of sessions delivered). This IF framework was determined and refined through discussions with the research team at the Leicester Diabetes Centre (LDC) and University of Leicester (UoL), the study team and findings from the feasibility study. IF will be achieved by training observers to observe the sessions. For each observation, the trained observers will complete a checklist supported by an IF coding manual which will be developed by the LDC/UoL team. The development process will include drafting the checklist and IF coding manual, testing them by carrying out inter-rater reliability and refining them until the level of agreement is reached.

#### Quantitative approach: data collection and analysis

Quantitative data will be extracted from Coach session logs, the Change One Thing app content management system, and the IF assessments to descriptively summarise:

- Number of sessions delivered: mean, SD; session length.
- Date of sessions (to derive session frequency).
- Mode of delivery (videocall, phone, in person): frequencies/percentages.
- List of intervention content areas with number (%) of participants who discussed each content area
- Average duration a participant stayed with the same action plan/content area
- Average number of intervention content areas covered during the total intervention period and in both the workbook and/or Change One Thing app.

#### Qualitative evaluation: recruitment, data collection and analysis

The research team at UoY will conduct semi-structured interviews/focus groups with participants, carers and DIAMONDS Coaches to determine engagement and satisfaction with the intervention. Interviews/focus groups will last approximately 45 min. The recruitment, data collection, and analysis for each of these cohorts are outlined below.

##### Participants

A sample of participants (20–25) will be approached by the research staff at the trusts on completion of the intervention and asked to provide written or verbal consent to take part in these 1–1 interviews. We aim to invite participants with a range of ages, genders, baseline health outcomes, comorbidities, levels of engagement with the workbook/app and levels of intervention completeness to inform sampling. These interviews/focus groups will explore participants’ experiences of intervention delivery and receipt, and any behavioural changes made to support their physical health and well-being.

##### Carers

The research staff at the trusts will be asked to identify, contact and recruit 20–25 carers for participation in the interviews. They will obtain either written or verbal consent. Only carers of service-users participating in the DIAMONDS RCT will be eligible. Once carers have given consent to the research team at the study site and permissions are in place to share contact details, this information will be passed on to the study team at UoY who will be responsible for arranging and conducting the interviews. Similar to the participant interviews, these will last approximately 45 min. For the purpose of this study, carers are defined as unpaid carers who are not subject to working regulations and provide support to a dependent person who they have a social relationship with, such as a spouse, other relative, neighbour, friend or other non-kin.

##### Coaches

On completion of their intervention sessions, all Coaches will be invited to take part in interviews/focus groups. Coach interviews are expected to last 30 min and will explore questions around the DIAMONDS Coach training, delivering the intervention, engagement with Coach support and barriers and enablers to implementing the intervention in existing health and care services.

All interviews/focus groups will be digitally recorded (with participant consent), anonymised and transcribed, with the transcripts forming the data for analysis. An initial thematic analysis^53^ will be conducted using a framework method.^54^ An initial coding framework will be developed, and transcripts checked against the framework to ensure that there are no significant omissions. Codes will be examined across individual transcripts as well as across the entire data set and allocated to the framework. Using aspects of the constant comparison method of analysis, broader categories using linking codes will be developed across the transcripts.

Further analysis will be guided by the MoA framework that extends the Theoretical Domains Framework (TDF).^55^ The TDF offers a robust theoretical basis for understanding implementation problems^56^ and has previously been used to frame the focus of a process evaluation of a behaviour change intervention.^57 58^

### Integrated analysis

A triangulation protocol will be used to explore opportunities to further integrate the quantitative and qualitative data. The sources of data will include IF assessments about adherence and quality of intervention delivery; patient participant and informal caregiver interview data about experiences of intervention receipt and Coach interviews/focus group data about experiences of intervention delivery. Key findings will be compared (in pairs) across the data sets using a convergence coding matrix. For each qualitative theme, we will investigate whether we can identify analogues in the quantitative data. We will then categorise the relationship between findings from the qualitative and quantitative data according to four categories: agreement (convergence in the data), partial agreement (complementary findings but limited overlap), silence (no overlap between quantitative and qualitative data) and dissonance (disagreement between data sets).

### Economic evaluation

The health economic analysis will take the form of a within-trial cost-utility analysis using an NHS and personal social services perspective as recommended by NICE guidance^59^ undertaken over a 12 month period. Additional details of the economic evaluation can be found in online supplemental appendix 7.

### Adverse event reporting, harms and participant withdrawals

#### Adverse events

An adverse event (AE) is any unexpected effect or untoward clinical event affecting the participant (ie, any unfavourable and unintended sign, symptom or disease). It can be directly related, possibly related or completely unrelated to the intervention. Any AEs or serious adverse events (SAEs) will be recorded by the R&D team at sites using specific AE/SAE forms. The reporting period will be from study entry to the last follow-up visit, and all events related to the DIAMONDS intervention will be recorded.

All SAEs are to be reported to the Chief Investigator and will be reviewed by a clinician independent of the DIAMONDS study team. All SAEs will be reported to the Sponsor and Research Ethics Committee (REC) in line with their guidelines. Ongoing review of AEs will take place during the Programme Management Team and PSC meetings.

#### Suicide and self-harm risk management

We have developed a suicide risk protocol for the monitoring of suicide and self-harm risk during all encounters with study participants. Where any risk to participants, due to expressed thoughts of self-harm or suicide is encountered, a risk assessment will be conducted. Prior to conducting the risk assessment, the participant will be advised that if there is a concern of risk of harm to themselves or others, concerns will need to be passed on to another party, such as their GP or clinical care team.

#### Duty of care

We will use YTU standard operating procedures to support researchers to report to GPs or responsible services instances where there are concerns about the health of the participant. Normal NHS indemnity procedures will apply as participants are recruited from NHS sites. The Sponsor (UoY) will also provide standard public liability insurance to meet the potential legal liability of the sponsor for harm to participants arising from the design and management of the research.

#### Researcher safety and lone working

We will use the YTU standard operating procedures/UoY Department of Health Sciences policy for fieldwork and lone working. All researchers tasked with fieldwork will undertake lone worker training and conduct a risk assessment with their line manager about the specific tasks to be carried out.

#### Participant withdrawals

Participants will be able to withdraw from the trial at any point without having to provide a reason and without it affecting their usual care or any benefits to which they are entitled. If a participant decides to withdraw, their quality of care will not be compromised.

The participant’s clinical team will also be able to withdraw participants if they lose capacity or become unfit to continue.

There are three categories of withdrawal: withdrawal from follow-ups, withdrawal from intervention (ie, withdrawal from engaging with Coaches and workbook) or full withdrawal. Where withdrawal is from intervention only, follow-up data will continue to be collected from the participant. Data provided by participants who decide to withdraw will be retained for analysis up until the point of withdrawal.

### Trial oversight

A Trial Management Group will monitor the day-to-day management of the trial. An independent PSC will have oversight of the trial and, due to the low-risk nature of this trial, it will also undertake the role of the Data Monitoring Committee.

### Ethics and dissemination

The study received ethical approval by the West of Scotland 3 (22/WS/0117). It is registered with the ISRCTN (ISRCTN22275538) and CPMS (53712). Since the approval of the trial there have been three modifications to the protocol. The current protocol is version 1.3 (07.02.2024).

We aim to publish the findings of the main study in peer reviewed, academic and professional journals to ensure that clinicians and academics have prompt access to our findings. We will produce a summary of the results that can be distributed to all trial participants and other relevant stakeholders (e.g. commissioners, third sector organisations) and will use social media channels, websites, and knowledge exchange events to communicate our findings beyond academic audiences. A publication policy has been agreed by the research team

## supplementary material

10.1136/bmjopen-2024-090295 online supplemental file 1
