# Supplementary material for: DIAMONDS—a diabetes self-management intervention for people with severe mental illness: protocol for an individually randomised controlled multicentre trial
Source: BMJ Open. 2025 Mar 27;15(3):e090295. doi: 10.1136/bmjopen-2024-090295 (PMC11956296; doi:10.1136/bmjopen-2024-090295)
Supplement: online supplemental file 1 [file bmjopen-15-3-s001.docx]

**Appendices**

**Appendix 1 – Full details of outcome measures**

Primary outcome

The primary outcome of the DIAMONDS programme is the difference in glycated haemoglobin (HbA_1c_) between the treatment groups at 12 months post-randomisation. To avoid the inadvertent introduction of differences in measurements of HbA_1c_ through the use of several local laboratories, we will arrange the use of one central United Kingdom Accreditation Service (UKAS) registered laboratory for all blood sample analyses. Blood samples will be sent to the lab from the participating sites. The lab will return test results (recorded as mmol/mol and %) to the study team at the UoY by post. Participants who decline a blood test will be asked to consent to sharing the results of their most recent routine blood test results held in primary or secondary care records. HbA_1c_ represents average blood glucose over a six to eight-week period. We will only use routine test results that have been reported between six weeks before to six weeks after the end of the scheduled follow-up date. We anticipate that the DIAMONDS intervention will lead to a number of behaviour/lifestyle changes, which directly or indirectly affect blood glucose levels. Glucose homeostasis is associated with cardio- and cerebrovascular events, microvascular complications and mortality in diabetes. We have, therefore, chosen a measure of glucose control, HbA_1c_, as our primary outcome and included a number of other important parameters that influence morbidity and mortality as secondary outcomes.

Secondary outcomes

*Physical health*

- Cholesterol: Measured as part of a biochemical lipid profile (blood test) taken at the same time as the blood for the HbA_1c_ measurement. Total cholesterol, HDL cholesterol, and triglycerides (all measured in mmol/L) will be recorded. LDL cholesterol will also be calculated.
- Haemoglobin: Blood test taken at the same time as blood for HbA_1c_ measurements and lipid profile. Haemoglobin will be recorded in g/L. All blood tests will be conducted at the same central laboratory. We will share participants’ blood results with their GP via post marked ‘confidential’.
- Body mass index (BMI): Calculated using weight (kg) and height (metres) measurements using the following formula: $\frac{weight (kg)}{{height (metres)}^{2}}$
- Waist circumference: Measured following standard health care provider procedures and recorded in cm.
- Blood pressure: Systolic and diastolic blood pressure measured following standard trust procedure and recorded in mmHg.
- Smoking status: Assessed through participant self-report: yes/no/never.
- Urinary albumin to creatinine ratio: Will be extracted from patients’ medical records as a measure of diabetic nephropathy.

*Physical Activity*

Physical activity will be measured using the International Physical Activity Questionnaire (IPAQ).^60^This instrument is a 7-item self-reported (short form) assessing physical activity in the last 7 days. Results will be reported in categories of activity levels (low, moderate, high).

In addition, we will provide all participants with wearable wrist accelerometers to obtain an objective measure of physical activity. Accelerometers will be given to participants at their baseline assessment and again at the six months follow-up time point, where they will be asked to wear the device continually for seven days. At the end of the seven-day period, they will be asked to return the device to the UoY team; this will be facilitated by the study team as needed. The UoY team will carry out all data download and device set-up. The R&D teams will receive instructions on how to activate the devices to start data collection.

Acceptability of wrist-worn accelerometers in this population has been confirmed in the STEPWISE study and the DIAMONDS feasibility study. We will convert accelerometery data into activity profiles to assess time spent (a) in a sedentary state, (b) doing mild activity, or (c) doing moderate (or high) intensity activity.

*Psychological health measures*

- Psychiatric symptoms: Assessed using the Brief Psychiatric Rating Scale (BPRS).^61^The BPRS assesses the level of 18 symptom constructs such as hostility, suspiciousness, hallucination, and grandiosity. Each symptom construct ranges from 1 (not present) to 7 (extremely severe).
- Depressive symptoms: Assessed using the Patient Health Questionnaire-9 (PHQ-9).^62^The questionnaire comprises nine items which are individually scored as 0 (not at all) to 3 (nearly every day) and then added to provide an overall score. The lower the overall score, the lower the severity of depression.

*Diabetes measures*

- Diabetes distress: Assessed using the Problem Areas in Diabetes (PAID) scale, a self-report measure of diabetes distress.^63^Each of the questionnaire’s 20 items are measured on a five-point scale from 0 (not a problem) to 4 (a serious problem). These scores are summed and multiplied by 1.25 to generate a total score out of 100.
- Summary of diabetes self-care activities: Assessed using the Summary of Diabetes Self-Care Activities Measure (SDSCA).^64^ This tool contains 11 items, which measure the frequency of performing diabetes self-care activities over the last seven days. The respondent marks the number of days on which the indicated behaviour was performed using an eight-point Likert scale. The first ten items are summed for a total score. Item 11 focuses on smoking habits and assesses the average number of cigarettes smoked per day.
- Insulin use: Assessed through participant self-report (yes/no).
- Diabetes complications: Extracted from medical records.
  - Microvascular: Retinopathy, Neuropathy, Nephropathy
  - Macrovascular: Myocardial infarction, Peripheral vascular disease, Stroke and Amputation, foot ulcers

*Quality of Life*

Health-related quality of life: Assessed using the EQ-5D-5L, a self-report measure.^65^ This generic, patient-reported outcome measure has five health domains (mobility; self-care; usual activities; pain/discomfort; and anxiety/depression) with five response options for each domain (no problems, slight problems, moderate problems, severe problems, and extreme problems). Responses are coded as single-digit numbers expressing the severity level selected in each dimension. For the economic evaluation, the profile of responses to the five domains will be converted to a utility value (see details in Appendix 4). In addition, it has a health status visual analogue scale (VAS) that measures self-rated health anchored at 0 (‘the worst health you can imagine’) and 100 (‘the best health you can imagine’).

*Health resource use*

A bespoke health resource use questionnaire that has been tested in the feasibility study and refined in line with feedback received will be used to collect participants’ use of primary care, secondary care and community-based services over a six-month period.

*Mechanisms of Action*

We will quantitatively collect information about the mechanisms of action (MoAs) used in the DIAMONDS intervention. The ‘Change One Thing’ app has built-in monthly reviews of MoAs that participants will work through with their DIAMONDS Coach at their 1-to-1 sessions. In addition, we will use a set of self-report process measures at baseline and follow-up.

**Appendix 2 – SPIRIT checklist**


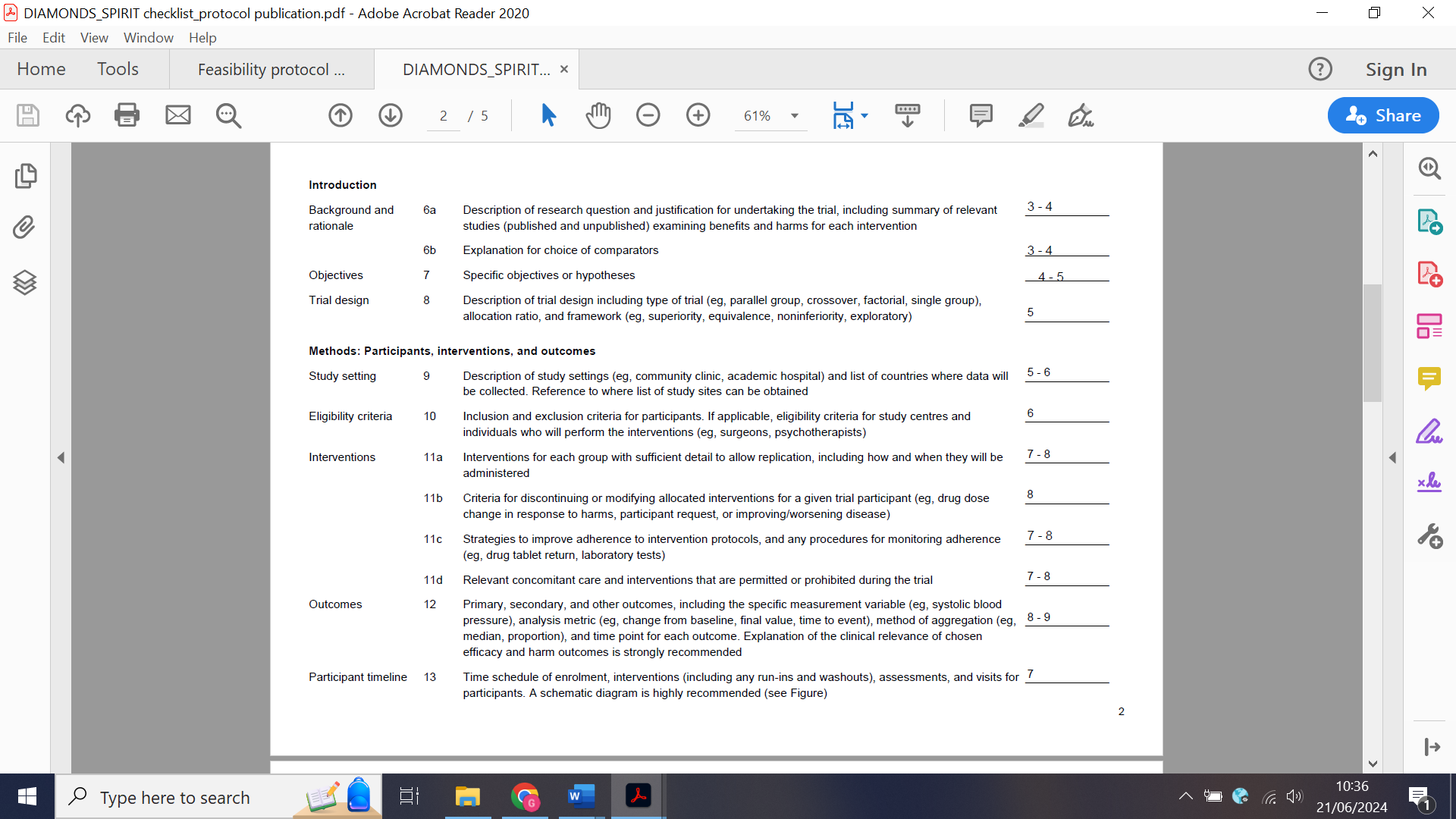

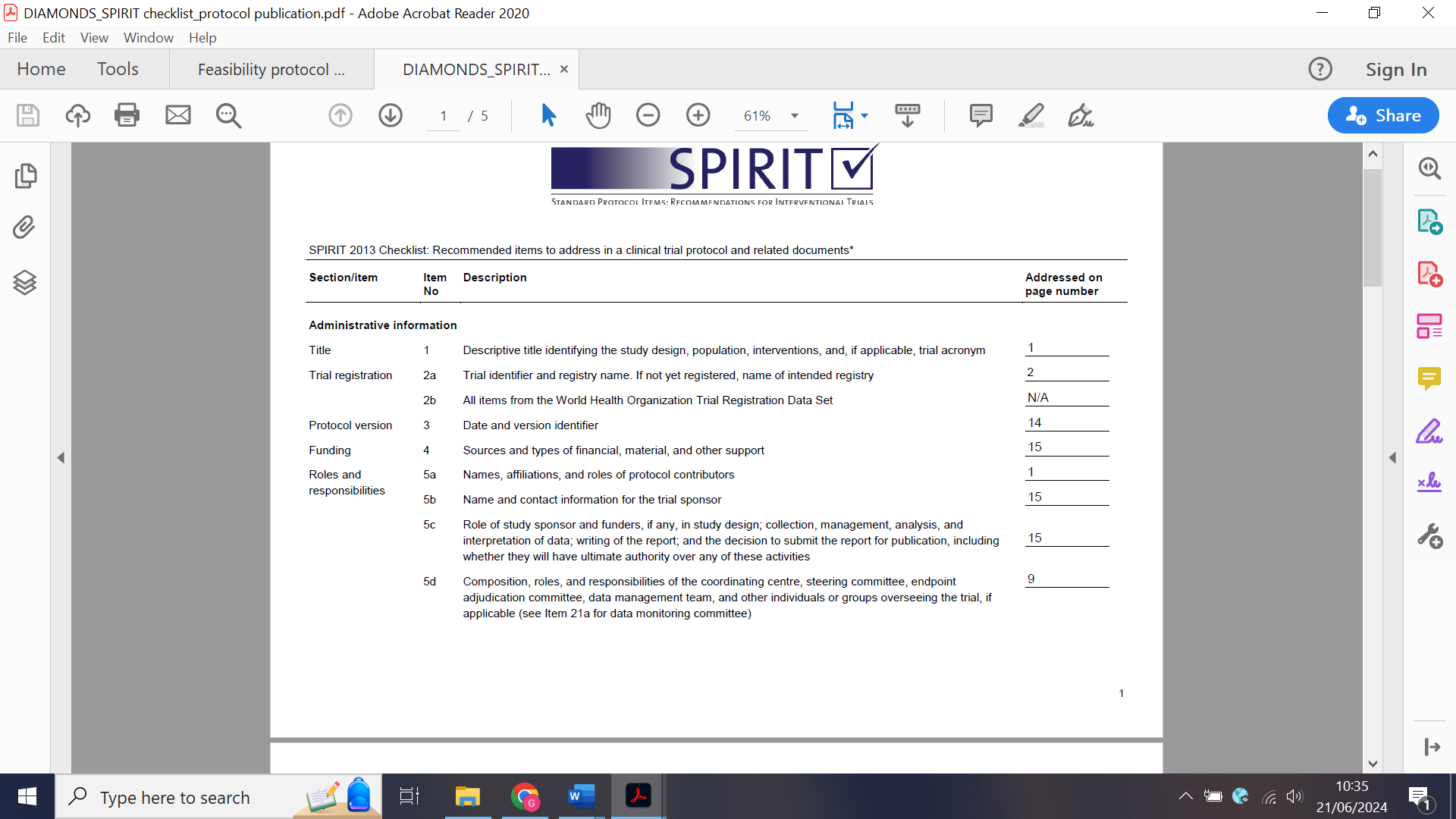


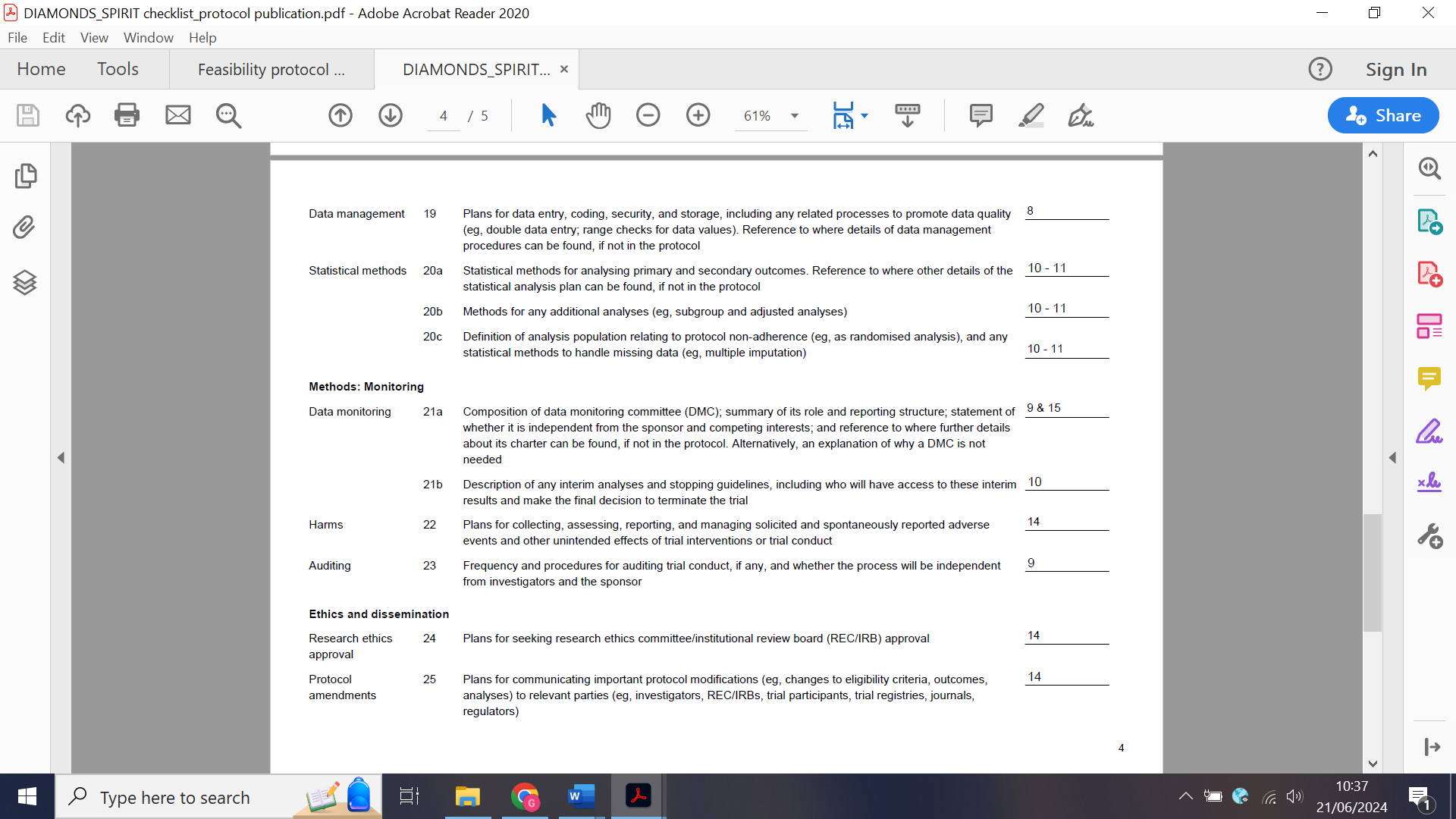

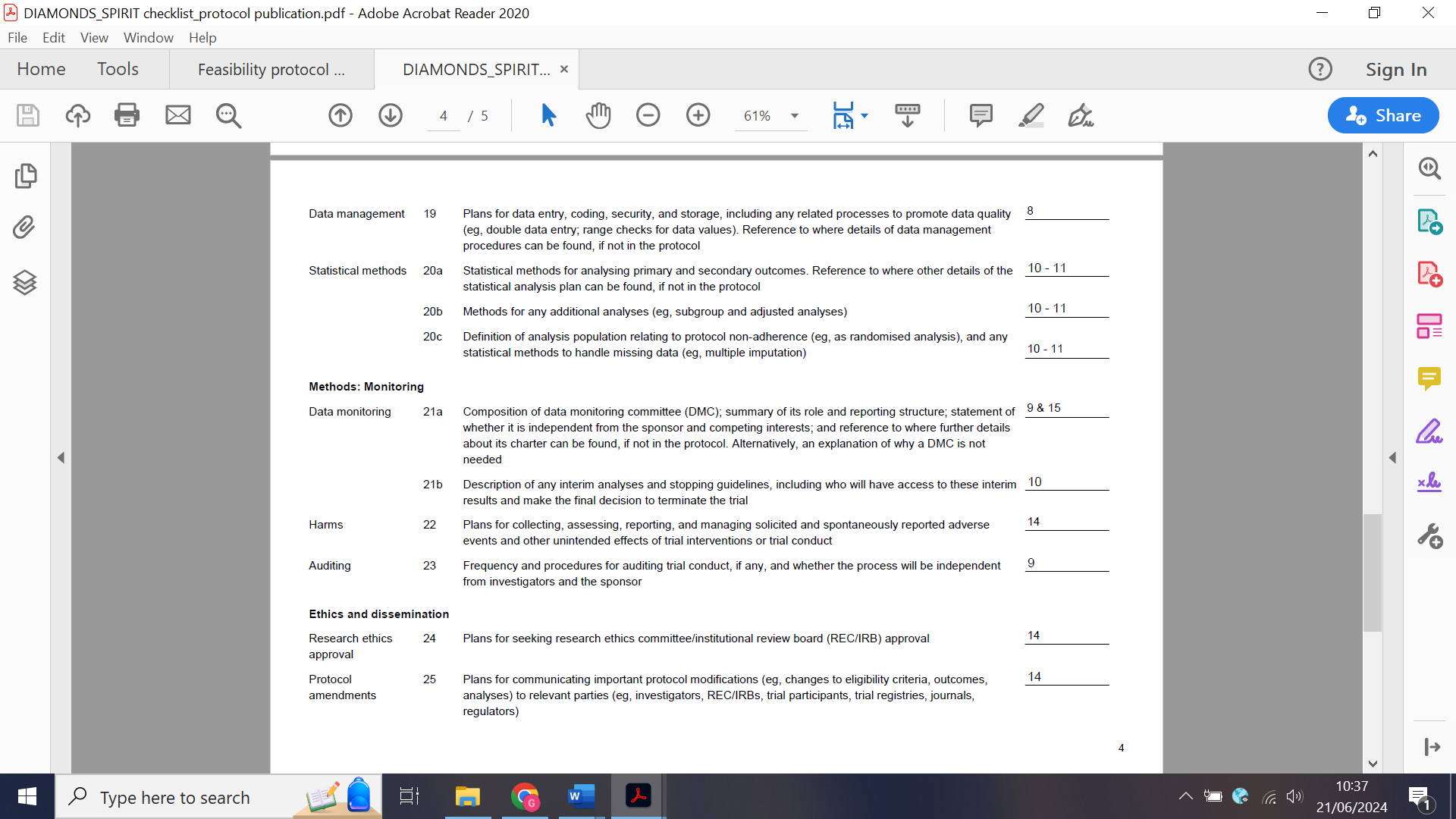

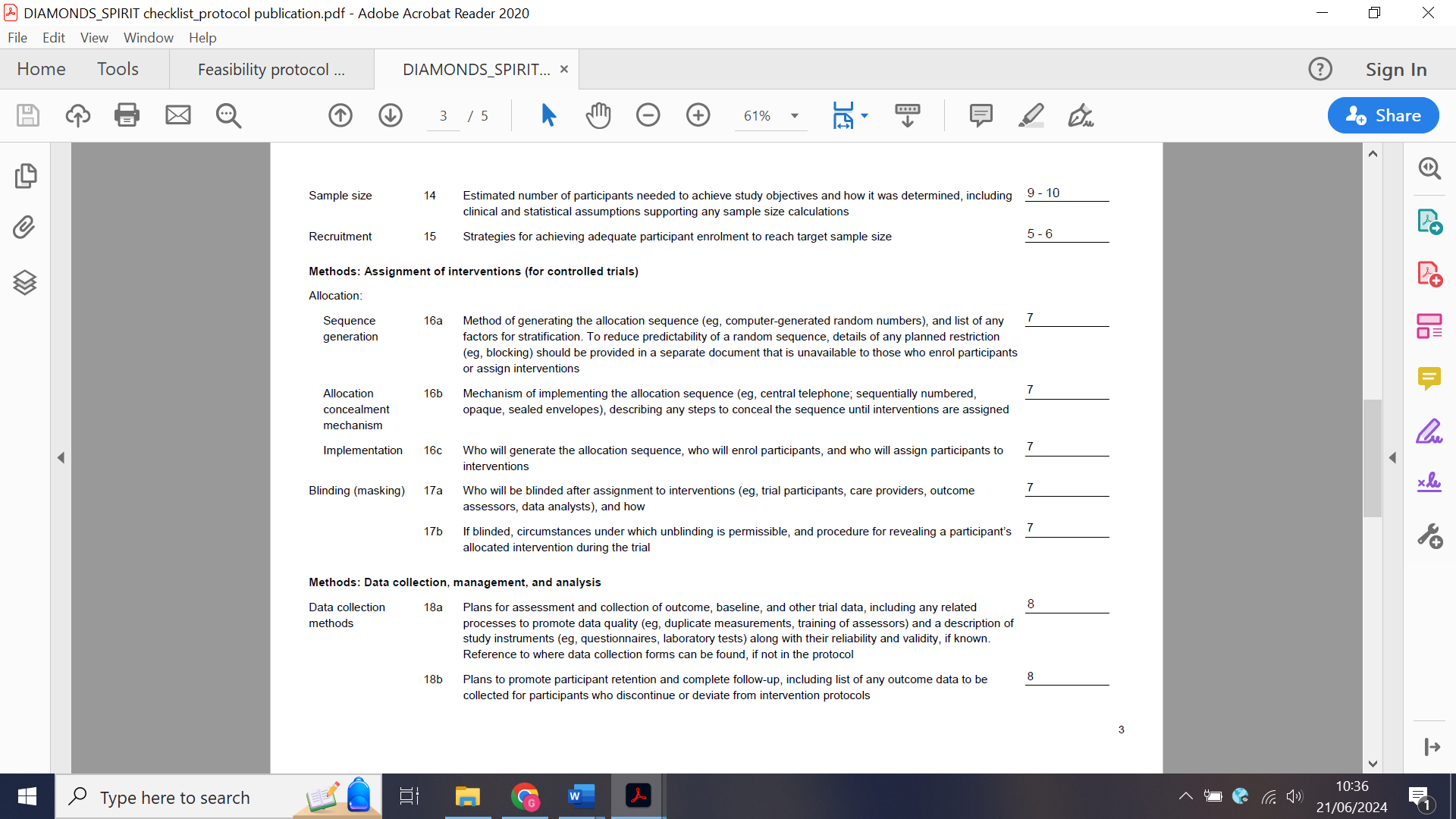


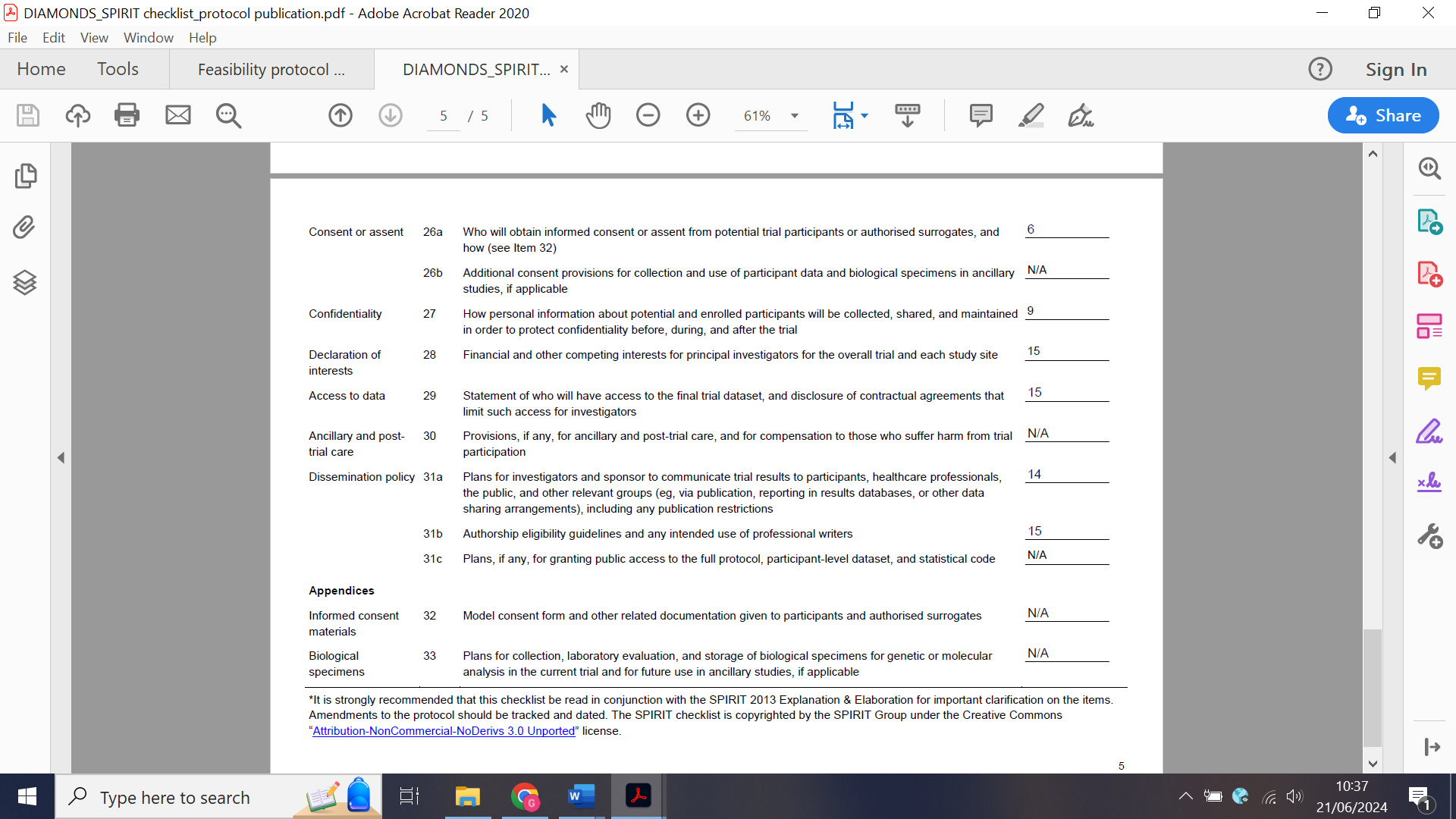


**Appendix 3 – Internal pilot**

The recruitment rate and 95% confidence interval (CI) will be estimated from the data collected. A CONSORT diagram will be produced to show the flow of participants through the study and the following outcomes calculated: number of eligible patients; proportion of eligible patients approached for consent; proportion of eligible patients not approached and reasons why; proportion of patients approached who provide consent; proportion of patients approached who do not provide consent; proportion of patients providing consent who are randomised; proportion of patients randomised who do not receive the randomly allocated treatment; proportion of patients dropping out between randomisation and follow-up.

Data will be summarised on the reasons why eligible patients were not approached, reasons for patients declining to participate in the study; reasons why randomised patients did not receive their allocated treatment and reasons for dropout, if available.

Results will be compared against the study’s recruitment assumptions and progression targets, and continuation of the trial or relevant modifications will be decided by the Steering Committee and the funding body.

Progression from the pilot phase to the main trial will depend on satisfying pre-specified targets at 12 months from the start of the trial:

|  | Green | Amber | Red |
| --- | --- | --- | --- |
| **a) Average number of participants per site per month** | 2 participant per month | 1.3 to <2 participant per month | <1.3 participant per month |
| **b) Recruitment of sites** | 15 sites | 10 to 14 sites | <10 sites |
| **c) Completeness of outcome (HbA_1c_) data at 6-months** | 80% of participants with complete outcome | 65% to <80% of participants with complete outcome | <65% of participants with complete outcome |

The actions taken for the progression criteria are outlined below:

- Green: continue the trial.
- Amber: review procedures to identify underlying problems, and put in place strategies to address these, review after an interval and terminate the trial if recruitment trajectory does not indicate that full recruitment will occur within scheduled recruitment period.
- Red: terminate the trial unless we can confidently identify successful strategies or rapidly resolve the problem.

**Appendix 4 – Consent form**

**
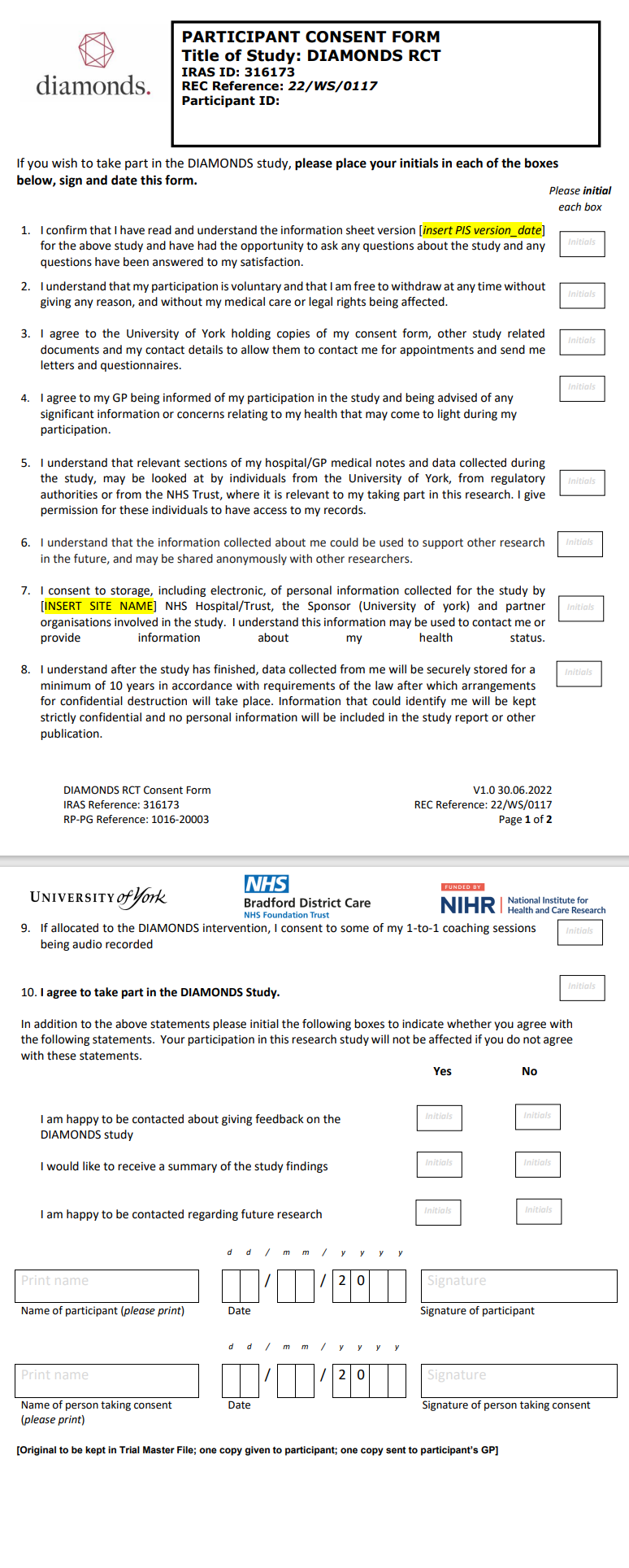
**

**
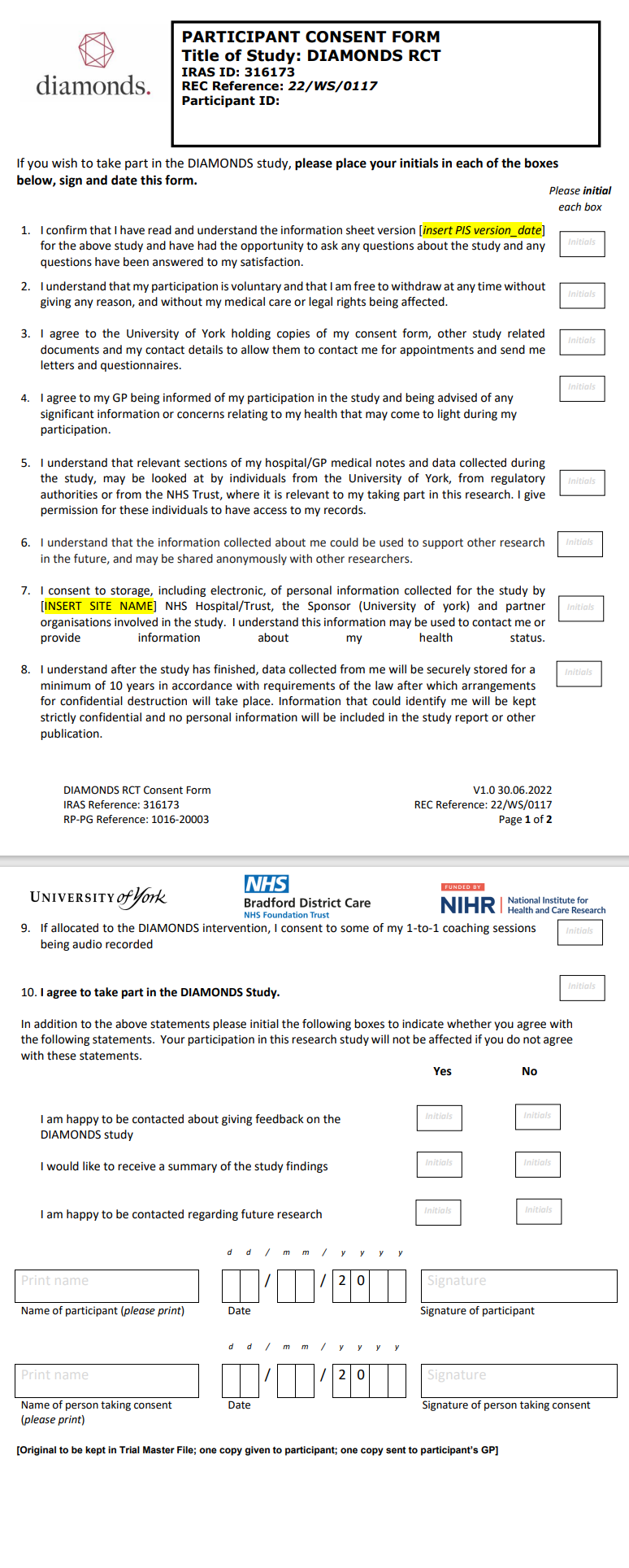
**

**Appendix 5 – Data collection table**

|  |  | Baseline | 6 months | 12 months |
| --- | --- | --- | --- | --- |
| Demographics | | | |  |
| Age | Self-report | x | - | - |
| Sex | Self-report | x | - | - |
| Ethnicity | Self-report | x | - | - |
| Index of Multiple Deprivation | Determined by study team based on participant’s postcode | x | - | - |
| Type of SMI | Medical records | x | - | - |
| Date diagnosed with SMI | Medical records | x | - | - |
| Date diagnosed with diabetes | Medical records | x | - | - |
| Physical Health | | | | |
| Height | Measured by study team | x | x | x |
| Weight | Measured by study team | x | x | x |
| BMI (calculated from height and weight) | Calculated by study team | x | x | x |
| Waist circumference | Measured by study team | x | x | x |
| Blood pressure | Measured by study team | x | x | x |
| HbA_1c_ | Measured by study team | x | x | x |
| Total and HDL cholesterol | Measured by study team | x | x | x |
| Haemoglobin | Measured by study team | x | x | x |
| Psychological health | | | |  |
| Brief Psychiatric Rating Scale (BPRS) | Self-report | x | x | x |
| Patient Health Questionnaire-9 (PHQ-9) | Self-report | x | x | x |
| Diabetes measures | | | |  |
| Diabetes distress (PAID) | Self-report | x | x | x |
| Summary of Diabetes Self-Care Activities | Self-report | x | x | x |
| Smoking status | Self-report | x | x | x |
| Physical activity (IPAQ) | Self-report | x | x | x |
| Diabetes microvascular and macrovascular complications | Medical records | x | x | x |
| Health economic outcomes | | | |  |
| Health-related quality of life (EQ-5D-5L) | Self-report | x | x | x |
| Health resource use | Self-report | x | x | x |
| Process evaluation measures | | | |  |
| Mechanisms of Action | Self-report | x | x | x |

**Appendix 6 – Data protection regulations**

*Data Protection:* The University of York complies with all aspects of the 2018 General Data Protection Regulation and Data Protection Act 2018. Operationally this will include obtaining explicit consent from study participants to record personal details including name, postal and email address, and contact telephone numbers; and appropriate storage, restricted access and disposal arrangements for their personal details. All participants will be informed of their rights in regard to the personal information stored, including erasure, rectification and objection. All work will be conducted following the University of York’s data protection guidance which is publicly available here <https://www.york.ac.uk/records-management/dp/>

*Data Security:*

- All data will be stored in accordance with data protection requirements and will be kept either in a locked filing cabinet in a secure office or in the case of electronic data on a secure server with a password protected computer and files.
- Personal addresses, postcodes and other contact details of consenting participants will be stored on a secure password-protected server located at the University of York, for the purposes of assisting in follow-ups during the study. All personally identifiable participant data will be coded, pseudonymised by participant number in all manual and electronic files. YTU will maintain a list of participant identification numbers for all trial participants at each site.
- Interview recordings will be downloaded onto a password protected computer and deleted from the recording device. They will then be securely uploaded to a GDPR-compliant transcribing company.
- No data will be stored on a home computer or laptop.
- All data will be stored for a minimum of 10 years, which will allow time for any academic challenge to be made. All data will be deleted after this time.

**Appendix 7 – Health Economics analysis**

Intervention costs will be collected throughout the trial to enable a bottom-up costing. We will record all costs incurred in the DIAMONDS Coach training stage and the intervention delivery stage. Training related activities, personnel, materials will be recorded as the training proceeds. The intervention session costs will be estimated based on the records of attendance of each participant. For the optional element that is the Change One Thing app, we will include the operational costs but not the development costs.

Quantities of wider health care use will be recorded by self-report questionnaires, as refined following the findings of the feasibility study. Unit costs will be taken from published secondary sources such as Unit Costs of Health & Social Care^66^ and NHS National Cost Collection (NCC)^67^, of the appropriate version at the time of the analysis. Unit costs will be applied to the quantities of care used to derive a cost profile for each participant in the trial.

Mean costs per participant will be presented for each trial group for intervention costs and also for wider health care use, broken down into individual care categories, with mean number of contacts and costs presented individually. Standard deviations are also presented alongside means.

We will collect EQ-5D-5L^65^ at baseline, 6- and 12-month follow-ups. The complete profile of five domains will be converted to a utility value using the method recommended by NICE^68^. These utility values will then be used to calculate Quality-Adjusted Life Years (QALYs), following the area under the curve approach^69^ using the three recorded time points. The QALY will be the primary outcome of effect for the economic evaluation.

Missing data patterns will be examined by treatment group, baseline characteristics, and values at prior time point. The results will inform the model of multiple imputation. The primary analysis of economic evaluation will be based on the imputed dataset.

Difference in costs and QALYs between treatment groups will be estimated using mixed effect regression models. Costs and QALYs will be the dependent variables and their respective baseline values, other baseline covariates will be used as fixed effects. The variation will be explored by adding the DIAMONDS Coach variable as a random effect. Costs, comprising treatment costs and wider NHS and costs, and QALY data will be combined to calculate the incremental cost-effectiveness ratio (ICER), if both incremental costs and QALYs are positive. The ICER will be compared to maximum acceptable threshold values to assess the value of money afforded by the intervention over and above the control. Underlying uncertainty will be assessed using non-parametric bootstrap re-sampling technique. Bootstrapping is an efficient method as the validity of uncertainty measures estimated via bootstrapping does not depend on any specific form of underlying distribution. We will generate 5,000 bootstrap replications and construct the 95% confidence intervals for incremental costs and QALYs respectively based on the bootstrapping results. Cost-effectiveness acceptability curves (CEACs)^70^, will be constructed based on the bootstrap iterations to estimate the probability that the intervention is cost-effective at different threshold values for one QALY.

To assess the impact of imputing missing data, we will also conduct a complete case analysis based on the participants who have both complete costs and QALYs at all timepoints, following the same analysis method as the primary analysis above. We will also conduct sensitivity analyses using pattern mixture modelling to examine the assumptions for multiple imputation methods^71^.

We will present costs based on the estimated number of patients in the total population who could receive and benefit from this intervention (i.e. beyond the trial), to provide a realistic per-patient estimate. We will estimate costs required to update and maintain the intervention to represent a realistic ‘roll out’ cost.

**Appendix 7 - Consent Form**
