# Supplementary material for: DIAMONDS—a diabetes self-management intervention for people with severe mental illness: protocol for an individually randomised controlled multicentre trial
Source: BMJ Open. 2025 Mar 27;15(3):e090295. doi: 10.1136/bmjopen-2024-090295 (PMC11956296; doi:10.1136/bmjopen-2024-090295)
Supplement: Supplementary file 2 [file bmjopen-2024-090295.reviewer_comments.pdf]

PEER REVIEW HISTORY

BMJ Open publishes all reviews undertaken for accepted manuscripts. Reviewers are asked to complete a checklist review form and are provided with free text boxes to elaborate on their assessment. These free text comments are reproduced below.

ARTICLE DETAILS

Title (Provisional)

DIAMONDS - A diabetes self-management intervention for people with severe mental illness: Protocol for an individually randomised controlled multi-centre trial.

Authors

O'Carroll, Grace Catherine; Brown, Jennifer V E; Carswell, Claire; Peck, Charlie; Russell, Gregor; Ajjan, RA; Boehnke, Jan Rasmus; Coventry, Peter A; Hadjiconstantinou, Michelle; Hewitt, Catherine; Holt, Richard Ian Gregory; Johnson, Vicki; Kellar, Ian; Li, Jinshuo; Mandefield, Laura; Osborn, David; Parrott, Steve; Sheehan, Lucy; Shiers, David; Watson, Judith; Siddiqi, Najma; Team, DIAMONDS Research

---

VERSION 1 - REVIEW

**Reviewer**	**1**
**Name**	**Chrzanowski, Jędrzej**
**Affiliation**	**Medical University of Lodz, Department of Biostatistics and Translational Medicine**
**Date**	**13-Aug-2024**
**COI**	**No competing interest to declare.**

I appreciate the opportunity to review the manuscript "DIAMONDS - A diabetes self-management intervention for people with severe mental illness: Protocol for an individually randomised controlled multi-centre trial." The manuscript describes in great detail a clinical trial protocol for the interventional clinical trial "Improving diabetes self-management for people with severe mental illness". This clinical trial address an important gap in research and policy of the support of people with SMI in management of type 2 diabetes. There is still a clear inequality and mutiple barriers in optimal management of diabetes in people with psychiatric comobiridities, and growing evidence that mental health and diabetes are intertwined (both in type 2 and type 1 diabetes). Overall, the manuscript is of a good quality, provides extensive details and clearly describes the interventions.

However, I have some suggestions that may improve the readibility of the manuscript:

1) Throughout the manuscript, a feasibility study is mentioned. It is not clear if that feasibility study is a part of the trial, or was it conducted and published before. Moreover, in the section: "The acceptability of the intervention to participants and DIAMONDS Coaches was confirmed in the DIAMONDS feasibility study. Prior to the start of the RCT, we refined the intervention in line with findings from the feasibility study which are reported elsewhere" - suggests that those results have been already published. If it is so, please provide the reference. If not, please revise.

2) In the manuscript, you specified that "an independent Programme Steering Committee will have oversight of the trial and due to the low risk nature of this trial, it will also undertake the role of the Data Monitoring Committee" but also that "R&D staff responsible for data collection remain blinded to treatment allocation" and "the DIAMONDS Programme Manager and Trial coordinators will remain un-blinded and will not be involved in analysis of data." This is quite diffucult to follow - are independent Programme Steering Committe/Data Monitoring Committee separete from R&D staff responsible for data collection and the DIAMONDS Programme Management and Trial coordinator? Please revise for clarity.

3) "To promote participant retention, each participant will be offered a £10 high street gift voucher at their baseline, 6- and 12-month appointments." Although the reimbursement is a common practice for the interventional clinical trials, the gifts to promote participant retention have been previously discussed in context that they might affect the informed consent. This might also rise some ethical concerns, especially for marginalized communities. Please, elaborate on this aspect and address this ethical issue.

4) Please elaborate on reporting and management of AE/SAE and SUSAR - specify the timing, stopping criteria and conditions, and who is responsible for monitoring of events.

5) Please specify the patient's path after withdrawal. You specified that "there are three categories of withdrawal: withdrawal from follow-ups, withdrawal from intervention, or full withdrawal" - do they affect the trial ending procedures differently?

6) Consider to provide access to model consent for and any other related documentation given to participants as per SPIRIT guideline.

7) Please provide relevant information on participants insurence within the interventional clinical trial as per SPIRIT guideline.

8) Please revise the figures, especially Figure 2. Currently, the Figure 2 seems pixelated - might be due to the submission system compression. Nonetheless, consider revising the figures to increase the font size if possible.

Reviewer **2**

Name **Stenager, Elsebeth**

Affiliation **Psychiatric Research Unit, Aabenraa, Department of Regional Health Services Research, University of Southern Denmark,**

Denmark; University of Southern Denmark, Odense, Denmark.; Department of Regional Health Research, University of Southern Denmark, Odense, Denmark., Department of regional Health Research

Date **13-Sep-2024**

COI **None**

This is a very well planned and well-organized study protocol concerning a diabetes self-management intervention for people with severe mental illness.

The protocol include a feasibility study and a RCT multicenter study. The RCT Study follow well known criteria's for RCT studies. The Spirit checklist has been used.

In general the author group have considered very many problems concerning planning of complex TCT study i.e. common laboratory facilities, relevant outcome measures , recruitment of patients from various sites, and blinding. The intervention is planned together with users, careers, and professionals, and the previous feasibility study is of great importance in the general planning. The control group is relevant and the treatment as usual described.

The intervention DIAMONDS is well described. In addition, the user involvement is an important part of the planning of intervention.

The sample size calculation seems relevant even though it had been adjusted, due to problems to involve a sufficient number of patients.

Because of the very good planning of the study I have only one major comment.

BMC demands, that dates of the study should be included in the manuscript. This is not the case neither for the fidelity study nor for the RCT study. It seems from the protocol that the RCT study is now ongoing, but the authors should include relevant dates for the study periods in the protocol.

VERSION 1 - AUTHOR RESPONSE

Reviewer 1 comments:

Response: Thank you for your positive comments regarding our manuscript. We have listed your specific points below, which we hope we have now addressed:

1. Throughout the manuscript, a feasibility study is mentioned. It is not clear if that feasibility study is a part of the trial, or was it conducted and published before. Moreover, in the section: "The acceptability of the intervention to participants and DIAMONDS Coaches was confirmed in the DIAMONDS feasibility study. Prior to the start of the RCT, we refined the intervention in line with findings from the feasibility study which are reported elsewhere" - suggests that those results have been already published. If it is so, please provide the reference. If not, please revise.

Response: Thank you. We have now included reference to the DIAMONDS feasibility study on page 3 and added a link on page 9 to the brief summary of findings that was submitted to the Health Research Authority.

2. In the manuscript, you specified that "an independent Programme Steering Committee will have oversight of the trial and due to the low risk nature of this trial, it will also undertake the role of the Data Monitoring Committee" but also that "R&D staff responsible for data collection remain blinded to treatment allocation" and "the DIAMONDS Programme Manager and Trial coordinators will remain un-blinded and will not be involved in analysis of data." This is quite difficult to follow - are independent Programme Steering Committee/Data Monitoring Committee separate from R&D staff responsible for data collection and the DIAMONDS Programme Management and Trial coordinator? Please revise for clarity.

   Response: Thank you for pointing this out. We have amended the layout and wording to try and improve clarity on page 8. We have also moved the paragraph regarding the Independent Programme Steering Committee (was page 10) to a new section entitled Trial Oversight (page 16).

3. "To promote participant retention, each participant will be offered a £10 high street gift voucher at their baseline, 6- and 12-month appointments." Although the reimbursement is a common practice for the interventional clinical trials, the gifts to promote participant retention have been previously discussed in context that they might affect the informed consent. This might also rise some ethical concerns, especially for marginalized communities. Please, elaborate on this aspect and address this ethical issue.

   Response: Thank you for the comment. We have updated the wording around participant retention on page 10. The inclusion of a voucher at 6 and 12 months was due to feedback from open, recruiting sites. This was submitted as an amendment and REC approval was received.

4. Please elaborate on reporting and management of AE/SAE and SUSAR - specify the timing, stopping criteria and conditions, and who is responsible for monitoring of events.

   Response: Thank you for this helpful comment. We have now amended this on page 15 and hope the process is much clearer now.

5. Please specify the patient's path after withdrawal. You specified that "there are three categories of withdrawal: withdrawal from follow-ups, withdrawal from intervention, or full withdrawal" - do they affect the trial ending procedures differently?

   Response: Thank you for the comment. We have now clarified this on page 16.

6. Consider providing access to model consent for and any other related documentation given to participants as per SPIRIT guideline.

   Response: Thank you for this suggestion. This is covered in the Editors comments.

7. Please provide relevant information on participants insurance within the interventional clinical trial as per SPIRIT guideline.

   Response: Thank you for this suggestion. We have added these details to page 16

8. Please revise the figures, especially Figure 2. Currently, the Figure 2 seems pixelated - might be due to the submission system compression. Nonetheless, consider revising the figures to increase the font size if possible.

   Response: Thank you for pointing this out. This was also noted by the Editor. This has now been addressed and our response has been provided in the Editor's comments.

Reviewer 2 comments:

Thank you for reviewing our manuscript and for your positive comments.

Response: We hope we have now addressed your specific comment:

1. BMC demands, that dates of the study should be included in the manuscript. This is not the case neither for the fidelity study nor for the RCT study. It seems from the protocol that the RCT study is now ongoing, but the authors should include relevant dates for the study periods in the protocol.

Response: Thank you for pointing this out. This was also noted by the Editor and the dates have now been added on page 6.

VERSION 2 - REVIEW

Reviewer 1

Name Chrzanowski, Jędrzej

Affiliation Medical University of Lodz, Department of Biostatistics and Translational Medicine

Date 21-Feb-2025

COI

My comments have been answered by the Authors and the manuscript has been revised appropriately. I have no further comments.
